# Comparing the Effects of Two Surfactant Administration Methods: Minimally Invasive Surfactant Therapy (MIST) with Intubation (INSURE) in Infants with Respiratory Distress Syndrome

**DOI:** 10.3390/arm92050036

**Published:** 2024-10-11

**Authors:** Hassan Boskabadi, Maryam Behmadi, Gholamali Maamouri, Tina Loghmani, Abdolrasoul Rangrazi

**Affiliations:** 1 Department of Pediatrics, Faculty of Medicine, Mashhad University of Medical Sciences, Mashhad 91388-13944, Iran; boskabadih@mums.ac.ir (H.B.); maamourigh@mums.ac.ir (G.M.); loghmanit4011@mums.ac.ir (T.L.); 2 Neonatal Research Center, Mashhad University of Medical Sciences, Mashhad 91388-13944, Iran; rangrazir@mums.ac.ir; 3 Dental Research Center, Mashhad University of Medical Sciences, Mashhad 91388-13944, Iran

**Keywords:** respiratory distress syndrome, surfactant, MIST, INSURE

## Abstract

**Highlights:**

**What are the main findings?**

- The MIST group showed a significantly reduced need for mechanical ventilation, as well as shorter durations of both CPAP and oxygen therapy compared to the INSURE group.
- Bronchopulmonary dysplasia (BPD) was also less frequent in the MIST group.
- There were no major differences between the two groups regarding other outcomes, such as hospital stay duration or complications like intraventricular hemorrhage (IVH), patent ductus arteriosus (PDA), necrotizing enterocolitis (NEC), pneumothorax, and pulmonary hemorrhage

**What is the implication of the main finding?**

- The MIST approach to surfactant therapy offers a practical, effective, and lower-risk alternative to the INSURE method.

**Abstract:**

Background: The aim of this study is to investigate and compare the effects of administering a surfactant through a fine intra-tracheal catheter during spontaneous breathing with the usual INSURE method in premature infants. Materials and Methods: In this clinical trial, premature babies with respiratory distress syndrome who required surfactant administration were randomly assigned to two groups: an intervention group (MIST) and a control group (INSURE). The treatment results were compared in terms of complications related to treatment (desaturation, apnea, bradycardia, and surfactant reflux), respiratory complications (requirement for mechanical ventilation, duration of nCPAP, duration of oxygen requirement, frequency of pneumothorax, and pulmonary hemorrhage), complications related to prematurity (incidence of IVH, NEC, BPD, and PDA), the need for a second dose of surfactant, and the duration of hospitalization. Results: a total of 160 premature babies with a gestational age of 26–34 weeks were randomly divided into two groups. The results showed that the need for mechanical ventilation, the duration of CPAP needed, and the duration of oxygen therapy were significantly lower in the MIST group than in the INSURE group. Additionally, the incidence of BPD was less common in the MIST group compared to the INSURE group. However, surfactant reflux was more common in the MIST group than in the INSURE group. There were no significant differences between the two groups in other outcomes, including the length of hospital stay and complications such as IVH, PDA, NEC, pneumothorax, and pulmonary hemorrhage. Conclusion: The results of this research demonstrate that the less invasive method of surfactant therapy (MIST) is a feasible, effective, and low-risk alternative to the INSURE method.

## 1. Introduction

Complications of preterm birth are the most common cause of death in children 5 years of age or younger. Major complications that lead to death in premature infants include respiratory distress syndrome (RDS), intraventricular hemorrhage (IVH), and necrotizing enterocolitis (NEC) [1]. RDS, caused by surfactant deficiency, is one of the main causes of respiratory failure, morbidity, and mortality in premature infants [2]. The incidence of RDS is inversely related to gestational age and birth weight, affecting 60% of infants born at less than 28 weeks of gestational age and 30% of those born between 28 and 34 weeks [3,4]. Before the introduction of surfactant therapy, RDS was considered the main cause of death in premature infants. Consequently, significant efforts have been made to prevent and treat RDS [5].

The preferred method for treating RDS is nasal continuous positive airway pressure (nCPAP) initiated at birth, along with the selective use of surfactant for infants with increasing oxygen needs. Surfactant administration can reduce the need for oxygen therapy, mechanical ventilation, air leakage syndrome, and mortality [6]. An exogenous surfactant is the most effective treatment for managing RDS because it improves pulmonary gas exchange in preterm infants by preserving functional residual capacity (FRC) and reducing the work of breathing [7].

Today, surfactant administration is typically performed using two methods. In the INSURE method (intubation, surfactant administration, and extubation), the neonate is intubated, administered surfactant, stabilized, and then extubated. In MIST (Minimally Invasive Surfactant Therapy), the surfactant is injected directly into the trachea via a thin catheter using laryngoscopy. During the common INSURE method, tracheal intubation sometimes fails, causing hypoxia, bradycardia, increased intracranial pressure, and mechanical ventilation-related damage, which can lead to barotrauma and chronic lung disease [6,8]. While this method prevents the complications of long-term mechanical ventilation, it still requires intubation, posing risks of trauma to the glottis and airways [9].

To achieve the benefits of surfactant while minimizing the complications of tracheal intubation, new methods of surfactant administration have been developed, allowing surfactant delivery without intubation. One such method involves administering the surfactant through a fine endotracheal catheter during the infant’s spontaneous breathing, which can significantly reduce surfactant administration complications [10].

Therefore, this study aims to compare the effects of administering surfactant through a fine intratracheal catheter during spontaneous breathing with the usual INSURE method in premature infants. Although studies on the MIST method exist in Iran and worldwide, research comparing the MIST and INSURE methods with a large sample size and more variables can provide a more comprehensive and complete view, helping to choose the best method for treating the critical complications of respiratory distress syndrome in infants.

## 2. Materials and Methods

### 2.1. Trial Design

This randomized clinical trial study was conducted on infants aged 26 to 34 weeks with respiratory distress syndrome at two educational, research, and treatment centers: Qaem (AS) and Imam Reza (AS) of Mashhad University of Medical Sciences. It spanned from May 2022 to September 2023.

The inclusion and exclusion criteria for study participants are as follows:

Inclusion criteria:

1. Newborns aged 26 to 34 weeks with respiratory distress receiving nasal continuous positive airway pressure (CPAP) via a T-Piece ventilator with a positive end-expiratory pressure (PEEP) set at 5–6 cm H_2_O at birth.
2. Diagnosis of RDS based on clinical and radiographic criteria.
3. Requirement for surfactant administration if FiO_2_ exceeds 30% despite receiving PEEP with a pressure greater than 6 cm H_2_O.

Exclusion criteria:

1. Presence of congenital anomalies.
2. Need for intubation and mechanical ventilation at birth.
3. Congenital infections.
4. Apgar score less than 5 at 10 min.
5. Congenital heart disease.

### 2.2. Interventions

**Figure 1:**
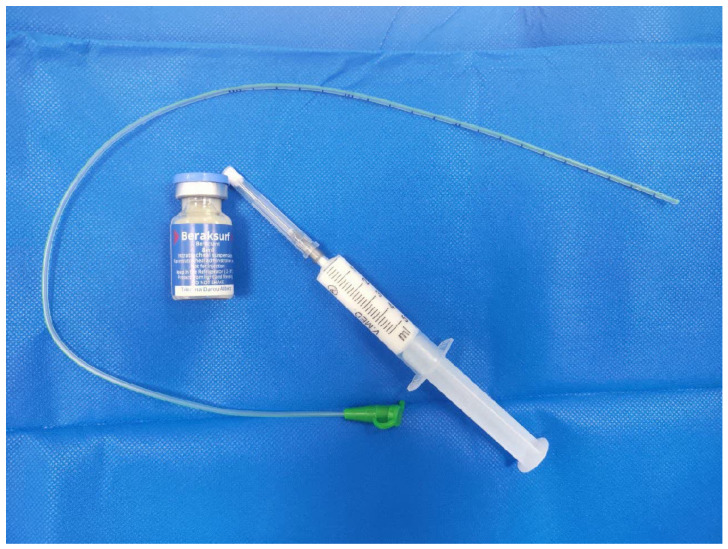
Feeding tube used in the MIST technique.

In the intervention group, premature infants received continuous positive airway pressure through nasal CPAP immediately after birth. If the need for FiO_2_ higher than 30% persisted despite PEEP of greater than 6 cm H_2_O [11], direct laryngoscopy was performed to administer the surfactant. A 6 French feeding tube was inserted into the infant’s trachea at a predetermined depth, securely fastened, and while the infant continued to breathe spontaneously under nCPAP, 100 mg/kg of the Beraksurf surfactant was administered through the feeding tube (Figure 1) over a period of 1–3 min. Upon completion of the surfactant injection, the feeding tube was removed and nCPAP continued.

In the control group, newborns were initially placed on nCPAP at birth. Subsequently, to administer the surfactant, nCPAP was discontinued and the infant was intubated with a tracheal tube of appropriate size to receive the same amount of surfactant through the tracheal tube. Once the infant was stabilized, the tracheal tube was removed within a few minutes, and nCPAP was resumed.

The severity of respiratory distress syndrome (RDS) was determined and compared based on scoring.

Throughout the procedures, the infant’s oxygenation status and heart rate were monitored via pulse oximetry. Bradycardia was defined as a heart rate of less than 100 beats per minute, desaturation as an SPO_2_ of 80% or less, and surfactant reflux as the visible return of surfactant to the mouth and nose or out of the tracheal tube. In both groups, if clinical or laboratory criteria indicative of respiratory failure, such as persistent apnea, pH less than 7.20, PCO_2_ greater than 65 mm Hg, or a significant reduction in SPO_2_, were observed at any time, the infant was intubated and placed on mechanical ventilation with a ventilator.

If FiO_2_ exceeded 40% and mean airway pressure (MAP) exceeded 7 cm H_2_O, a second dose of surfactant was administered using the same method as before [12].

For surfactant administration, we often used non-pharmacological and analgesic methods, including proper positioning and oral sucrose according to the Newborn Individualized Developmental Care Assessment Program (NIDCAP). In a small number of cases in both groups, intravenous fentanyl was administered as needed.

In both groups, venous blood gas (VBG) tests were conducted before and two hours after surfactant administration, and changes were evaluated and compared between the two groups. Following completion of the procedural steps, demographic information regarding the mother and baby, along with necessary data, were recorded and analyzed using pre-designed questionnaire forms.

### 2.3. Outcomes

The measured outcomes included respiratory outcomes such as the need for mechanical ventilation, the duration of nasal continuous positive airway pressure (nCPAP), the duration of oxygen therapy, and the occurrence of pulmonary hemorrhage and pneumothorax (based on clinical examination and X-ray). Treatment-related complications, including desaturation, apnea, bradycardia, and surfactant reflux, were also assessed. Additionally, prematurity-related complications during hospitalization were measured and recorded in both groups and compared. These complications included the frequency of intraventricular hemorrhage, the occurrence of patent ductus arteriosus (PDA) requiring treatment as determined by a pediatric cardiologist, and bronchopulmonary dysplasia (BPD) as defined by the most recent National Institute of Child Health and Human Development (NICHD) criteria [12]. The length of hospitalization in the Neonatal Intensive Care Unit (NICU) was also recorded.

### 2.4. Randomization

The blocking method was employed as a reliable randomization technique to ensure a balanced allocation of participants across treatment groups at the conclusion of each block. The website https://www.sealedenvelope.com/simple-randomiser/v1/lists (accessed on 25 May 2022) was utilized to generate random blocks with variable sizes. For this study, block sizes of 6 and 8 were chosen, resulting in a total of 13 blocks with size of 6 and 11 blocks with size of 8. Subsequently, each block was sealed in a non-transparent envelope, and all blocks were randomly arranged. Upon the referral of each eligible patient, the first block was randomly selected. Depending on the sequence order, the patient was then assigned to one of the study arms. This process continued sequentially, with subsequent blocks randomly selected and patients assigned until the last block.

### 2.5. Blinding

Upon the arrival of a newborn, a nurse randomly selected one of the sealed blocks without knowledge of the grouping. Subsequently, one of the numbers from the selected block was presented to the attending doctor, who was aware of the group allocation. The doctor then performed the assigned intervention based on the presented information. Following the intervention, the Neonatal Fellowship team monitored the baby for treatment complications and subsequent patient processes.

Data from the study were recorded in SPSS software (Version 26; IBM Corp, Armonk, NY, USA) using designated groups A and B. Importantly, the individual analyzing the data remained unaware of the specific intervention or control group assignments, ensuring impartial analysis.

### 2.6. Sample Size Calculation

Based on the study by Abdel-Latif et al. [13] and using a formula for comparing two proportions related to a qualitative trait from two populations, considering an alpha level of 0.05 and a beta level of 0.20 (80% power), it was determined that a minimum of 79 infants in each group would be required. Ultimately, 160 infants were included in the study.

### 2.7. Statistical Analysis

Data were collected, recorded, and entered into SPSS statistical software. To compare the mean of quantitative variables at different time points between the studied groups, a two-sample independent *t*-test or the non-parametric Mann–Whitney test was used. The Pearson Chi-Square test (or Fisher’s exact test, when appropriate) was used to compare qualitative variables between the two study groups. A significance level of *p* ≤ 0.05 was considered in all cases.

## 3. Results

**Figure 2:**
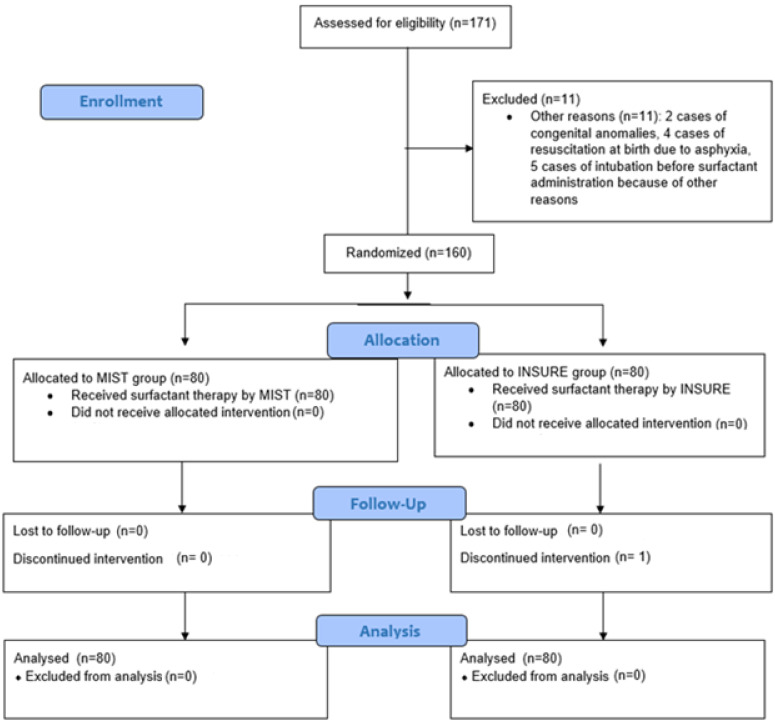
Consort flow chart.

During the specified period, a total of 171 infants were deemed eligible for the study, of whom 11 were excluded based on the study criteria (Figure 2). Consequently, 160 infants, with 80 infants in each group, were included and analyzed.

In our study, 86% of infants in the MIST group and 82% in the INSURE group were born by cesarean section (*p* = 0.514). Additionally, 56% of infants in the MIST group and 52% in the INSURE group were boys (*p* = 0.634). Other characteristics of infants before the intervention are summarized in Table 1.

Examining the treatment-related complications in the two studied groups revealed that although surfactant reflux was more common in the MIST group than in the INSURE group, no significant difference was observed in terms of other complications (Table 2).

In terms of respiratory complications, the results indicated that the need for mechanical ventilation (*p* < 0.001), the duration of nCPAP (*p* = 0.026), and the duration of oxygen therapy (*p* = 0.010) were significantly lower in the MIST group compared to the INSURE group. The average duration of mechanical ventilation was 11.66 h in the MIST group and 29.64 h in the INSURE group (*p* = 0.02). The average time of surfactant administration was 4.56 h in the MIST group and 3.85 h in the INSURE group, with no significant difference between the two groups (*p* = 0.361). Additionally, no significant difference was observed between the two groups in terms of the occurrence of pneumothorax (*p* = 0.468) and pulmonary hemorrhage (*p* = 0.405) (Table 3).

The results of our study indicated that there was no significant difference between the two groups in terms of the need for a second dose of surfactant (*p* value = 0.373). In terms of the incidence of BPD, a significant difference was observed between the two groups. Specifically, five cases of severe BPD were observed in the INSURE group, while no cases of severe BPD were observed in the MIST group (*p* value = 0.028). However, there was no significant difference between the two groups in terms of other complications (Table 4).

The average hospitalization period in the NICU for infants in the MIST group was 18.01 days, while in the INSURE group, it was longer at 20.24 days. However, there was no significant difference between the two groups in this regard (*p* value = 0.287). The duration to reach full feeding was investigated and compared between the two groups. Although the average time was 8.9 days in the MIST group and 10.7 days in the INSURE group, there was no significant difference between the two groups (*p* = 0.071).

Based on the results of this study, there was no significant difference between the MIST and INSURE groups in the levels of pH, HCO_3_, and PCO_2_ before surfactant administration. However, after surfactant administration, a significant difference was observed between the two groups. Specifically, the increase in blood pH after MIST surfactant administration was significantly higher than after INSURE (*p* value = 0.020). Nevertheless, no significant difference was observed between the two groups in terms of HCO_3_ and PCO_2_ levels (Table 5).

## 4. Discussion

The results of our study showed that infants treated with MIST needed a shorter duration of non-invasive respiratory support (nCPAP). The studies by Kanmaz et al. [14], Patra et al. [15], and Jena et al. [16] also confirm our findings. However, in the studies by Boskabadi et al. [17] and Kaleem et al. [18], no significant difference was observed between the two groups in terms of the duration of CPAP need. Additionally, in the multicenter study by Mirnia et al. [19], in contrast to our results, the duration of CPAP increased in the MIST group at one center [19].

The need for recurrent mechanical ventilation in infants treated with MIST in our study was lower than in the INSURE group. This finding is supported by the studies conducted by Anand et al. [20], Jena et al. [16], Dargaville et al. [21], Kanmaz et al. [14], Kaleem et al. [18], Wang et al. [22], and Boskabadi et al. [17] However, the studies by Choupani et al. [23] and Mohammadizadeh et al. [24] did not show any difference in this regard.

In our study, the duration of oxygen therapy in infants treated with the MIST method was less than that of the INSURE group. This finding is supported by the studies conducted by Gopel et al. [25], Jena et al. [16], Akhila et al. [26], and Mohammadizadeh et al. [24]. However, the studies by Choupani et al. [23] and Kaniewska et al. [27] did not show any difference in this regard.

The respiratory benefits of the MIST technique, including a reduction in the need for mechanical ventilation, CPAP duration, and oxygen therapy, might be explained based on animal studies demonstrating that even a small number of mechanical breaths are sufficient to cause lung injury in the neonatal period [28]. Additionally, the MIST method allows for the continuation of CPAP, thereby maintaining FRC and preventing atelectotrauma to the premature lung [29]. Moreover, it enables the baby to breathe spontaneously, facilitating the rapid and complete distribution of surfactant in the premature lung. Conversely, ventilation with positive pressure, as prescribed in the INSURE method, with non-homogeneous time constants, results in non-uniform lung expansion and increases the risk of velotrauma [16,30,31].

The results of our study showed that there is no significant difference between the MIST and INSURE groups in terms of the need to receive a second dose of surfactant. This finding is consistent with most studies, including those by Jena et al. [16], Anand et al. [20], Kaleem et al. [18], Akhila et al. [26], Choupani et al. [23], and Mirnia et al. [19]. However, in the studies by Garib et al. [32] and Mosayebi et al. [33], the need for a second dose of surfactant was higher in the MIST method.

In our study, treatment-related issues such as desaturation, bradycardia, and apnea did not show significant differences between the two groups. However, the occurrence of surfactant reflux was higher in the MIST group. This finding aligns with the studies by Mirnia et al. [19], Kanmaz et al. [14], and Anand et al. [20], which reported higher instances of surfactant reflux in the MIST group. Conversely, studies by Mosayebi et al. [33] and Yang et al. [34] found no difference between the two groups. Sabzehei et al. [6] reported more cases of reflux in the INSURE group, in contrast with our findings.

Surfactant reflux is a common side effect of surfactant administration using the MIST method. This may be attributed to the smaller diameter of the catheter compared to the tracheal tube, which may not completely fill the larynx, allowing the surfactant to return to the oral cavity spontaneously. Additionally, variations in the experience and skill level of the staff performing the procedure could contribute to the higher rate of reflux in the MIST group. Proper catheter placement and injection speed also play crucial roles in the occurrence of this phenomenon.

Furthermore, the use of a thin catheter may result in slower surfactant administration. Administering larger amounts of surfactant, such as Braksurf, may require more time. Therefore, employing surfactant products with smaller volumes and adhering to sufficient time according to the protocol could potentially mitigate this issue [35].

In Yang et al.’s study, similar to our findings, there was no significant difference between the MIST and INSURE groups in terms of bradycardia and desaturation [34]. However, in the studies by Jena et al. [16] and Patra et al. [15], bradycardia and desaturation were more prevalent in the MIST group compared to the INSURE group.

In terms of respiratory complications such as pneumothorax and pulmonary hemorrhage, no significant difference was observed between the two groups in our study. This lack of significant difference in terms of pneumothorax between the MIST and INSURE groups was also reported in other studies, including those by Anand et al. [20], Patra et al. [15], Kaniewska et al. [27], and Mirnia et al. [19].

No difference in pulmonary hemorrhage between the MIST and INSURE groups was observed in several studies, including those by Boskabadi et al. [17], Kaniewska et al. [27], Kaleem et al. [18], and Sabzehi et al. [6].

In our study, there was no significant difference between the two groups in terms of complications related to prematurity, including IVH, PDA, and NEC. This finding aligns with several studies, including those by Jena et al. [16], Anand et al. [20], Boskabadi et al. [17], and Kaleem et al. [18], which found no difference in terms of IVH between the MIST and INSURE groups.

Similarly, no significant difference in terms of PDA was observed between the two groups in several studies, including those by Jena et al. [16], Anand et al. [20], Garib et al. [32], Kaniewska et al. [27], and Mirnia et al. [36].

After the administration of surfactant, a significant portion of lung atelectasis is resolved, leading to improved oxygen delivery and reduced pulmonary vascular resistance. However, this may also increase the likelihood of a left-to-right shunt in the ductus arteriosus, potentially justifying the development of PDA complications in treated infants [37].

In terms of NEC, other studies such as those by Anand et al. [20], Garib et al. [32], Kaniewska et al. [27], and Mirnia et al. [19] also reported no significant difference between the MIST and INSURE groups.

In our study, a significant improvement in pH was observed over time after the MIST intervention, which could be attributed to the increase in HCO_3_ leading to a decrease in CO_2_ levels and a reduced need for supplemental oxygen. This finding is consistent with similar observations in the studies by Jaba et al. [38] and Nagwa et al. [39]. Additionally, Mirnia et al. [19] found a statistically significant increase in HCO_3_ levels two hours after surfactant administration in the MIST group.

One of the significant complications of RDS is BPD, which was reported to be less prevalent in the MIST method. This reduction in BPD in the MIST group could be attributed to the decrease in the need for invasive ventilation, a major risk factor for BPD [40]. Studies conducted on premature animal models indicate that even a few ventilation breaths administered immediately after birth can trigger the VILI cascade, which is one of the most crucial risk factors for BPD in premature infants [12]. Conversely, the higher incidence of BPD in the INSURE group could be attributed to damage resulting from intubation and short periods of positive pressure ventilation during surfactant administration. Additionally, it is believed that spontaneous breathing facilitated by the MIST technique distributes the surfactant more evenly with less damage, thus reducing the occurrence of BPD [33,41]. Similar to our findings, a decrease in the occurrence of BPD in the MIST group has been observed in other studies, including those by Jena et al. [16], Wang et al. [22], and Kanmaz et al. [14]. Conversely, no difference in terms of BPD between the two groups was reported in the studies conducted by Anand et al. [20], Boskabadi et al. [17], Kaleem et al. [18], and Choupani et al. [23].

## 5. Conclusions

In conclusion, the results of this research show that the less invasive method of surfactant administration (MIST) reduces the need for reintubation, the duration of oxygen therapy, and the duration of CPAP requirement, without increasing the incidence of prematurity-related complications compared to the standard INSURE method. Additionally, it lowers the risk of developing BPD in premature infants with respiratory distress syndrome. Therefore, the use of this method for surfactant injection is strongly recommended.

## Figures and Tables

**Table 1:** Baseline characteristics of two groups.

Group Characteristics	MIST	INSURE	*p* Value
Gestational age (weeks)	32.21 ± 1.94	31.58 ± 2.24	0.062
Birth Weight (gr)	1936.06 ± 581.03	1770.88 ± 579.99	0.069
First minute Apgar score	8 (4–9)	7 (4–9)	0.243
Fifth minute Apgar score	9 (6–10)	9 (6–10)	0.449
Mother Corticosteroid	59 (74%)	58 (72%)	0.858
Gestational Diabetes Mellitus (GDM)	5 (6%)	6 (6%)	0.063
Premature Rupture of Membranes (PROM) ≥ 18 h	13 (16%)	21 (26%)	0.122
Caesarean Section	69 (86%)	66 (82%)	0.514
Severity of RDS (RDS Score)	7.9 ± 1.1	8.1 ± 1.24	0.258
pH before surfactant	7.23 ± 0.06	7.24 ± 0.08	0.604

**Table 2:** Treatment complications of two groups.

	Group	MIST	INSURE	*p* Value
Treatment Complications				
Desaturation		42 (52%)	40 (50%)	0.752
Bradycardia		10 (12%)	10 (12%)	1
Reflux Surfactant		39 (49%)	20 (25%)	0.002
Apnea		3 (4%)	2 (2%)	1

**Table 3:** Respiratory complications of two groups.

	Group	MIST	INSURE	*p* Value
Respiratory Complications				
nCPAP Duration (Days)		3.57 ± 2.96	5.03 ± 5.01	0.026
O_2_ Therapy		9.13 ± 8.82	12.36 ± 11.95	0.01
Mechanical ventilation		6 (7%)	22 (27%)	0.001
Pneumothorax		3 (4%)	5 (6%)	0.468
Pulmonary hemorrhage		2 (2%)	4 (5%)	0.405

**Table 4:** Prematurity-related complications of two groups.

	Group		MIST	INSURE	*p* Value
Complications					
PDA			21 (26%)	29 (36%)	0.172
IVH			5 (6%)	12 (15%)	0.077
BPD		NO	76 (95%)	72 (90%)	0.028
		Mild	3 (4%)	0 (0%)	
		Moderate	1 (1%)	3 (4%)	
		Severe	0 (0%)	5 (6%)	
NEC			1 (1%)	2 (2%)	0.056
Late-onset sepsis			6 (7%)	7 (9%)	0.0772

**Table 5:** Venous blood gas analysis of two groups.

	Group	MIST	INSURE	*p* Value
VBG Factors				
pH before surfactant		7.23 ± 0.06	7.24 ± 0.08	0.604
HCO_3_ before surfactant		18.06 ± 2.89	19.12 ± 4.21	0.066
PCO_2_ before surfactant		47.82 ± 9.34	50.65 ± 14.29	0.142
pH after surfactant		7.33 ± 0.06	7.31 ± 0.06	0.029
HCO_3_ after surfactant		19.13 ± 2.73	19.51 ± 2.92	0.405
PCO_2_ after surfactant		38.45 ± 7.47	40.69 ± 9.49	0.101

## Data Availability

Data are contained within the article.
